# The 5-Lipoxygenase Inhibitor Zileuton Protects Pressure Overload-Induced Cardiac Remodeling via Activating PPAR*α*

**DOI:** 10.1155/2019/7536803

**Published:** 2019-11-03

**Authors:** Qing-Qing Wu, Wei Deng, Yang Xiao, Jiao-Jiao Chen, Chen Liu, Juan Wang, Yankai Guo, Mingxia Duan, Zhulan Cai, Saiyang Xie, Yuan Yuan, Qizhu Tang

**Affiliations:** ^1^Department of Cardiology, Renmin Hospital of Wuhan University, Wuhan 430060, China; ^2^Cardiovascular Research Institute, Wuhan University, Wuhan 430060, China; ^3^Hubei Key Laboratory of Cardiology, Wuhan 430060, China; ^4^Department of Cardiology, The Fifth Affiliated Hospital of Xinjiang Medical University, Ürümqi, China; ^5^Department of Pacing and Electrophysiological, First Affiliated Hospital of Xinjiang Medical University, Urumqi, Xinjiang 830011, China

## Abstract

Zileuton has been demonstrated to be an anti-inflammatory agent due to its well-known ability to inhibit 5-lipoxygenase (5-LOX). However, the effects of zileuton on cardiac remodeling are unclear. In this study, the effects of zileuton on pressure overload-induced cardiac remodeling were investigated and the possible mechanisms were examined. Aortic banding was performed on mice to induce a cardiac remodeling model, and the mice were then treated with zileuton 1 week after surgery. We also stimulated neonatal rat cardiomyocytes with phenylephrine (PE) and then treated them with zileuton. Our data indicated that zileuton protected mice from pressure overload-induced cardiac hypertrophy, fibrosis, and oxidative stress. Zileuton also attenuated PE-induced cardiomyocyte hypertrophy in a time- and dose-dependent manner. Mechanistically, we found that zileuton activated PPAR*α*, but not PPAR*γ* or PPAR*θ*, thus inducing Keap and NRF2 activation. This was confirmed with the PPAR*α* inhibitor GW7647 and NRF2 siRNA, which abolished the protective effects of zileuton on cardiomyocytes. Moreover, PPAR*α* knockdown abolished the anticardiac remodeling effects of zileuton in vivo. Taken together, our data indicate that zileuton protects against pressure overload-induced cardiac remodeling by activating PPAR*α*/NRF2 signaling.

## 1. Introduction

Cardiac remodeling refers to changes in the shape, structure, and function of the heart caused by pathophysiological stimuli [1]. Cardiac hypertrophy is one of the major responses to stress and/or myocardial damage and is characterized by increased myocardial cell volume and increased ventricular wall thickness [2]. Gradually, chronic stress or disease will lead to ventricular dilation and contractile function failure and eventually progress to heart failure. The signaling involved in cardiac remodeling includes mitogen-activated protein kinase (MAPK), AKT (serine/threonine kinase), Ca^2+^-calcineurin, and peroxisome proliferator-activated receptors (PPARs) [1, 3]. Among these signaling molecules, PPARs are of great importance. PPARs are significant in the regulation of carbohydrate and lipid metabolism in cells. Currently, three PPAR isoforms have been found, PPAR*α*, PPAR*β*/*δ*, and PPAR*γ* and they are encoded by separate genes [4]. All of these isoforms participate in the transcriptional regulation of multiple processes and are essential in the pathogenesis of cardiovascular diseases [4]. Thus, targeting PPAR molecules may be a promising therapeutic method for treating HF.

Zileuton, a well-known inhibitor of 5-LOX (catalyzes the conversion of arachidonic acid to leukotriene (LT)), has been proven as an anti-inflammatory drug [5]. Zileuton is a clinical drug that is already used in patients with chronic asthma [6] and chronic obstructive pulmonary disease [7]. Recently, a study reported the cardioprotective effects of zileuton, namely, good protective effects against H_2_O_2_-induced oxidative stress and cell damage in both cardiac myogenic H9c2 cells, and neonatal rat cardiomyocytes (NRCMs) were found [8]. Zileuton has been reported to protect against myocardial infarction injury in a rat model [9]. Zileuton was also found to inhibit the development of pulmonary hypertension in rats [10]. All of these studies indicate the protective effect of zileuton against cardiovascular disease, especially its direct effect on cardiomyocytes. A previous study found a link between 5-LOX and PPARs in neuroblastoma [11] and macrophages [12]. In this study, we explored whether zileuton could protect against cardiac remodeling upon pressure overload and the underlying mechanism involved in cardiomyocyte signaling under pathological conditions.

## 2. Materials and Methods

### 2.1. Animals

Animal operations were performed according to the Guide for the Care and Use of Laboratory Animals published by the US National Institutes of Health (NIH Publication No. 85-23, revised 1996) and were approved by the Animal Care and Use Committee of Renmin Hospital of Wuhan University. We purchased male C57/BL6 mice 8 to 10 weeks old from the Laboratory Animal Science Institute (Beijing, China). The animals were assigned randomly to 4 groups: the vehicle-sham group (*n* = 12), zileuton-sham group (*n* = 12), vehicle-AB group (*n* = 12), and zileuton-AB group (*n* = 12). The administration of zileuton (MedChemExpress, NJ 08852, USA, oral, once daily, 100 mg/kg) was started 1 week after AB surgery and maintained for 7 weeks. Mice also received a myocardial injection of AAV9-shPPAR*α* to knockdown PPAR*α* 2 weeks before surgery [13] in an aseptic surgery room (*n* = 12 in each group). Eight weeks after surgery, the mice were sacrificed, and the hearts were removed from all groups. Six hearts were fixed in formalin for histological analysis, and the other 6 hearts were placed in liquid nitrogen and then stored at -80°C until RNA and protein extraction.

### 2.2. AAV9 Construction and Viral Delivery

Vigene Biosciences (Shanghai, China) constructed AAV9-shPPAR*α* and negative shRNA (AAV9-shRNA). We purchased PPAR*α* siRNA from Santa Cruz (SC-422360). Two weeks prior to AB surgery, randomly selected mice received an AAV9-shPPAR*α* or AAV9-shRNA myocardial injection of 1 × 10^11^ vp (virion particles) per animal. Briefly, after the mice were anesthetized with 3% sodium pentobarbital (80 mg/kg, ip), the hearts of the mice were exposed and the pericardium was removed. We injected the following areas with a #29 syringe: the anterior wall, the side walls, and the apex of the left ventricle. A single needle (10 *μ*l) was inserted at the apex of the heart, and the two needles were inserted at the anterior wall and the side wall. The total amount of each adenoviral vector injection (1 × 10^11^ vp) was 50 *μ*l, and the injection interval was approximately 5 mm.

### 2.3. Echocardiography and Hemodynamics

Echocardiography was performed using a MyLab 30CV ultrasound (Biosound Esaote) as previously described [14, 15].

Cardiac hemodynamics were determined using a microtip catheter transducer (SPR-839; Millar Instruments, Houston, TX) as previously described [14, 15].

### 2.4. Histological Analysis and Immunohistochemistry

As described in a previous study [14, 15], the mice were sacrificed 8 weeks after surgery and the hearts were removed from all groups (*n* = 6), placed in 10% formalin, and then embedded in paraffin. Hematoxylin and eosin (HE) staining was used to detect the cell transverse area, which was visualized by light microscopy. Image-Pro Plus (version 6.0) was used to quantitate the digital images. A total of 100 to 200 myocytes were counted in each heart section. For immunohistochemistry, hearts from all groups (*n* = 6) were incubated with anti-CD45 (ab25603, Abcam), anti-CD68 (ab125212, Abcam), or 4-hydroxynonenal (4-HNE, ab46545, Abcam) antibodies. The cells were then incubated with goat anti-rabbit EnVision™+/horseradish peroxidase (HRP) reagent, and a DAB detection kit was used for coloration.

### 2.5. RT-PCR and Western Blot Analyses

Eight weeks after surgery, the mice were sacrificed and the hearts were removed from all groups (*n* = 6), placed quickly in liquid nitrogen, and then stored at -80°C. RNA from left ventricle (LV) tissue and cells was isolated, purified, and then reverse transcribed into cDNA using oligo (DT) primers and a Transcriptor First-Strand cDNA Synthesis Kit. PCR amplification was conducted with a LightCycler 480 and SYBR® Green 1 Master Mix. GAPDH was used as the reference gene.

For Western blotting, protein was isolated and quantified from LV tissue (for all groups, *n* = 6) and cells. Then, the protein samples (50 *μ*g) were loaded onto 10% SDS-PAGE gels. The following primary antibodies were used: SOD2, NADPH P67, gp91, PPAR*γ*, PPAR*α*, PPAR*ƍ*, keap1, PPAR*γ* coactivator 1*α* (PGC1*α*), nuclear respiratory factor 2 (NRF2), lamin B (purchased from Abcam), and GAPDH (purchased from Santa Cruz). After incubation with the appropriate secondary antibody, the blots were scanned using a two-color infrared imaging system (Odyssey, LI-COR, Lincoln, NE, USA). GAPDH was used as the reference protein.

### 2.6. Cell Culture

H9c2 cells were purchased from the Cell Bank of the Chinese Academy of Sciences (Shanghai, China). Zileuton was dissolved in DMSO at a concentration of 1 mM. Phenylephrine (PE) (Sigma) (50 *μ*m) was added to the medium in the presence or absence of different concentrations of zileuton (1, 10, 50, and 100 *μ*m), and the cells were cultured in this medium for 24 h. To knockdown Nrf2, cells were treated with Nrf2 siRNA (Santa Cruz, SC-156128). To inhibit PPAR*α*, cells were treated with GW6471 (10 *μ*M, MedChemExpress). To knockdown 5-LOX, cells were transfected with either 5-LOX siRNA (5′-GCAAGAGGACCTCATGTTT-3′) (RiboBio, Shanghai, China) or control Sc-RNA for 8 h.

### 2.7. Oxidative Stress

SOD, glutathione peroxidase (Gpx), and NADPH oxidase in cardiac tissue and cardiomyocyte lysates were detected by commercial kits (Beyotime Biotechnology, China). ROS levels were detected by a DCFH-DA probe and a fluorescence microplate reader.

### 2.8. Cell Viability and Cell Surface Area

Cell viability was detected using a commercial MTT kit (Beyotime Biotechnology, China) according to the manufacturer's instructions. An anti-actinin antibody (05-384, Millipore) was used to stain the cells according to our previous study [16]. Image-Pro Plus (version 6.0) was used to determine the surface areas of single cells.

### 2.9. Statistical Analysis

The data are expressed as the mean ± standard deviation. Comparisons between the two groups were analyzed using two-way analysis of variance, followed by a post hoc Tukey test. We used Student's unpaired *t*-test to compare the differences between two groups. A *P* value of less than 0.05 (two tailed) was considered statistically significant.

## 3. Results

### 3.1. Zileuton Ameliorates Cardiac Hypertrophy and Improves Cardiac Function in Response to Pressure Overload In Vivo

**Figure 1:**
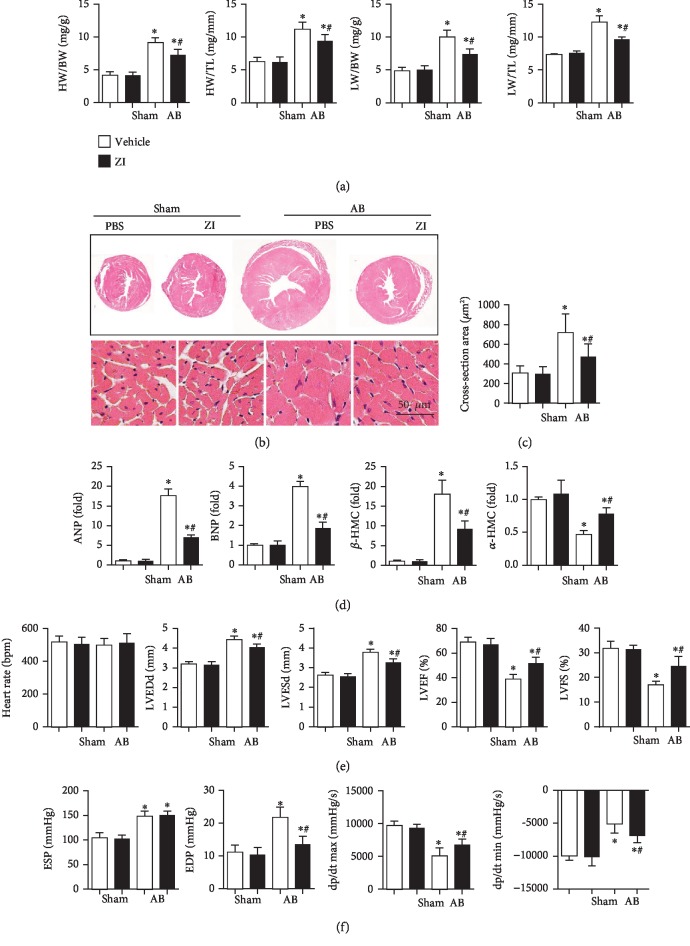
Zileuton ameliorates cardiac hypertrophy and improves cardiac function in response to pressure overload in vivo. Mice were administered zileuton orally (100 mg/kg/d) from 1 week until 8 weeks after AB surgery. (a) Heart weight/body weight (HW/BW), heart weight/tibia length (HW/TL), lung weight/body weight (LW/BW), and lung weight/tibia length ratios in mice (*n* = 12). (b, c) HE staining (*n* = 6) and quantification results in mice (*n* > 50 cells). (d) Transcription levels of hypertrophic markers (*n* = 6). (e) Echocardiography in mice (*n* = 10). (f) Hemodynamic measurements in mice (*n* = 10). ^∗^*P* < 0.05 vs. the vehicle-sham group; ^*#*^*P* < 0.05 vs. the vehicle-AB group. ZI: zileuton.

To explore the protective role of zileuton against cardiac hypertrophy, aortic banding was performed on mice to induce a cardiac hypertrophy model. Zileuton was given 1 week after surgery and continued for 7 weeks. No difference in cardiac function was found between the two AB experimental groups (Table 1). A remarkable hypertrophic response was observed in mice that were subjected to AB surgery, as assessed by increased HW/BW, HW/TL, LT/BW, and LW/TL ratios. The cross-section area and the transcription levels of hypertrophic markers were increased in the mouse hearts from the AB group. Zileuton dramatically inhibited these hypertrophic responses, as all of these indexes were reduced when compared to those in the vehicle-AB group (Figures 1(a)–1(d)). Echocardiography and pressure-volume loop analyses were used to detect cardiac function. The heart rate was not significantly different between the AB and sham groups. After 8 weeks of AB surgery, the mice exhibited ventricular dilation and systolic and diastolic dysfunctions as evidenced by increased LVESd and LVEDd, reduced LVEF and LVFS, elevated end-systolic pressure (ESP) and end diastolic pressure (EDP), and decreased maximal rate of pressure development (dp/dt max) and maximal rate of pressure decay (dp/dt min) (Figures 1(e) and 1(f)). Zileuton ameliorated LV dilation and cardiac dysfunction as evidenced by decreased LVEDd, LVESd, and EDP and increased LVEF, LVFS, dp/dt max, and dp/dt min. There were no differences in the ESP between the vehicle- and zileuton-treated groups after AB (Figures 1(e) and 1(f)).

### 3.2. Zileuton Relieves Inflammation and Oxidative Stress In Vivo

**Figure 2:**
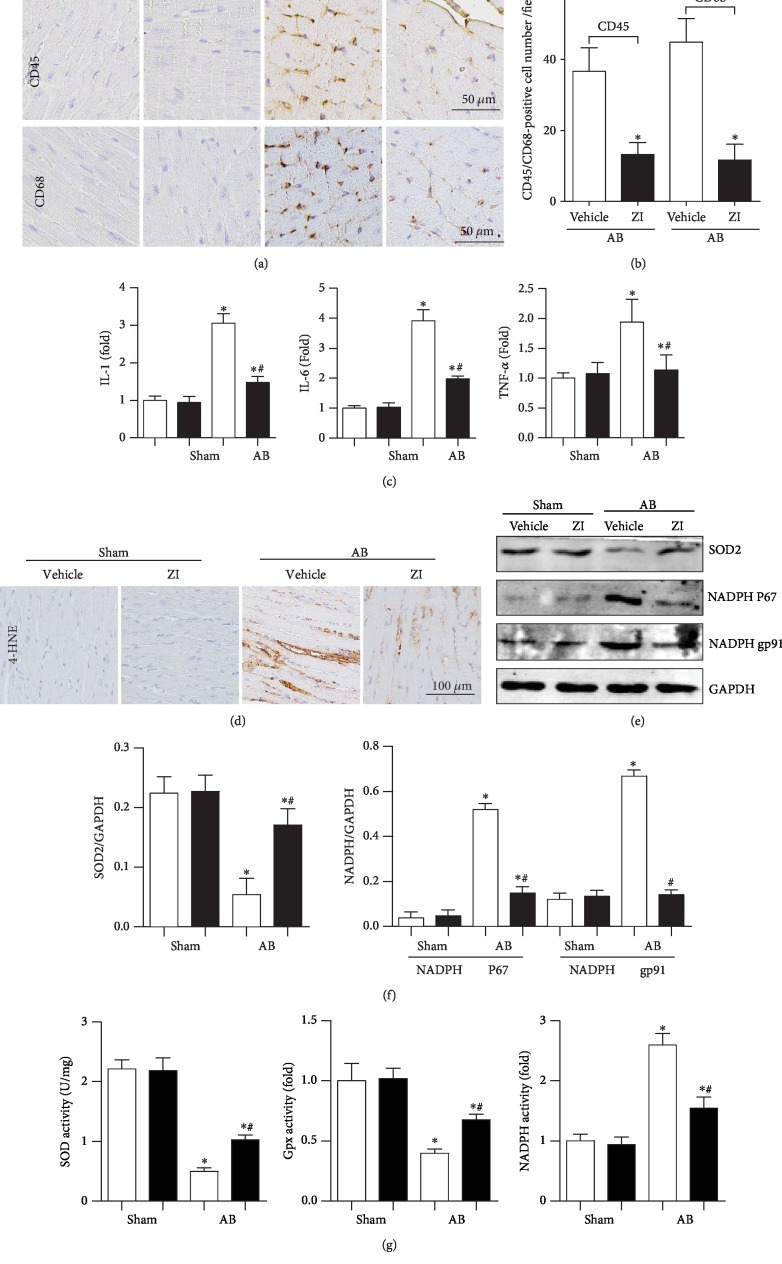
Zileuton relieves inflammation and oxidative stress in vivo. Mice were administered zileuton orally (100 mg/kg/d) from 1 week until 8 weeks after AB surgery. (a, b) Immunohistochemical staining and quantification results in mouse hearts (*n* = 6). (c) Transcription levels of inflammation markers (*n* = 6). (d) Immunohistochemical staining of 4-HNE (*n* = 6). (e, f) Protein expression levels of SOD2, NADPH P67, and gp91 (*n* = 6). (g) Activities of SOD, Gpx, and NADPH oxidase (*n* = 6). ^∗^*P* < 0.05 vs. the vehicle-sham group; ^*#*^*P* < 0.05 vs. the vehicle-AB group.

Cardiac inflammation is one of the characteristics of heart failure. Thus, we detected the inflammatory response. As shown in Figure 2(a), CD45-labeled leukocytes and CD68-labeled macrophages were increased in the AB group. The transcription of proinflammatory markers (TNF-*α*, IL-1, and IL-6) was also increased in the AB group. Zileuton treatment reduced the infiltration of CD45-labeled leukocytes and CD68-labeled macrophages and decreased the transcription of those inflammatory markers (Figures 2(a)–2(c)).

We next detected oxidative stress in failing mouse hearts. As shown in Figure 2(d), the intermediate metabolites of lipid peroxidation, including 4-HNE, were increased in the hearts of AB surgery mice. The expression and activity of the antioxidase SOD were decreased, and the expression and activity of the oxidase NADPH P67 and gp91 were increased in AB mouse hearts. The activity of Gpx was also decreased in the hearts of AB surgery mice. Zileuton treatment ameliorated these conditions, as evidenced by reduced production of 4-HNE, increased expression and activities of SOD and Gpx, and reduced expression and activity of NADPH oxidase (Figures 2(d)–2(g)).

### 3.3. Zileuton Reduces Cardiomyocyte Hypertrophy in Response to PE

**Figure 3:**
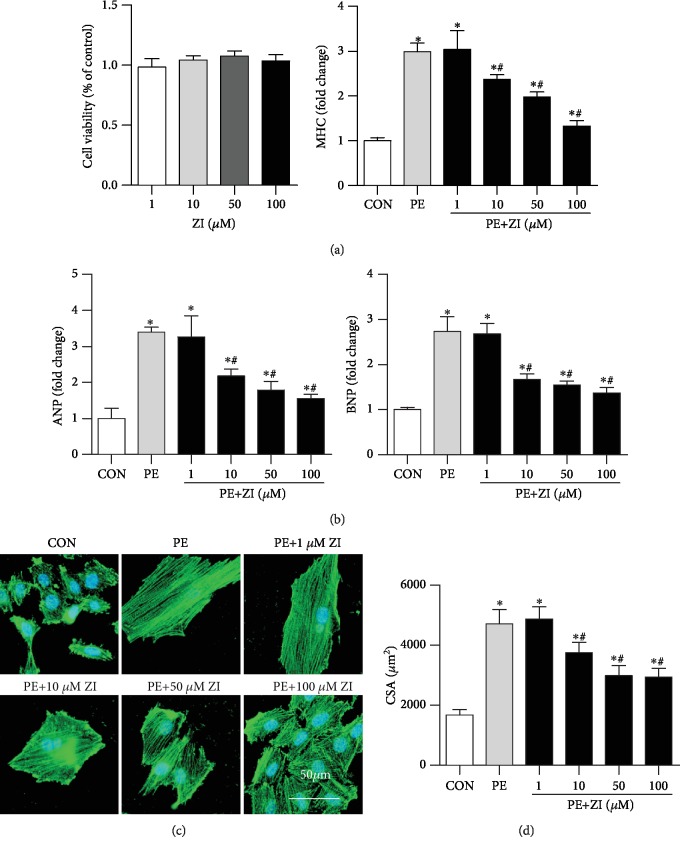
Zileuton reduces cardiomyocyte hypertrophy in response to PE. H9c2 cells were treated with PE (50 *μ*M) and different concentrations of zileuton (1, 10, 50, and 100 *μ*M) for 24 h. (a) Cell viability (*n* = 6). (b) Transcription levels of hypertrophic markers in H9c2 cells (*n* = 6). (c, d) *α*-Actinin staining (*n* = 6) and quantification of cells. ^∗^*P* < 0.05 vs. the control group; ^*#*^*P* < 0.05 vs. the PE group.

To explore whether the effect of zileuton on cardiac hypertrophy relied on cardiomyocytes, H9c2 cardiomyocytes were cultured and treated with different concentrations of zileuton. The results showed that zileuton (1, 10, 50, and 100 *μ*M) did not affect cell viability (Figure 3(a)). Cells were stimulated with PE for 24 h to establish a cardiomyocyte hypertrophy model. Zileuton (10, 50, and 100 *μ*M) exerted an antihypertrophy effect on H9c2 cells in a dose-dependent manner, which was evidenced by the reduced transcription level of hypertrophic markers (ANP, BNP, and *β*-MHC) and the decreased cell surface area induced by PE stimuli (Figures 3(b)–3(d)). Thus, we chose 100 *μ*M zileuton for further study.

### 3.4. Zileuton Inhibits Oxidative Stress in Cardiomyocytes

**Figure 4:**
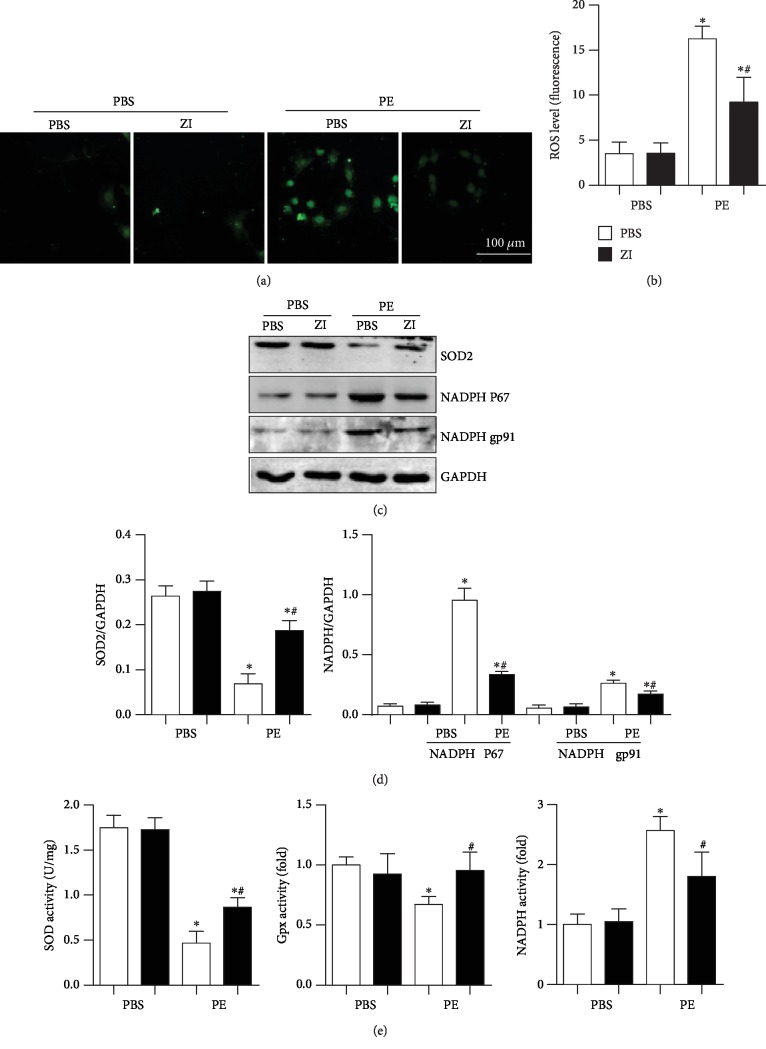
Zileuton inhibits oxidative stress in cardiomyocytes. H9c2 cells were treated with PE (50 *μ*M) and zileuton (100 *μ*M) for 24 h. (a, b) ROS levels in H9c2 cells (*n* = 6). (c, d) Protein expression levels of SOD2, NADPH P67, and gp91 (*n* = 6). (e) Activities of SOD, Gpx, and NADPH oxidase (*n* = 6). ^∗^*P* < 0.05 vs. the PBS group; ^*#*^*P* < 0.05 vs. the PE group.

The antioxidative stress effects of zileuton on cardiomyocytes were detected. As shown in Figure 4, the ROS level was increased in PE-stimulated cardiomyocytes. The expression and activity of the antioxidase SOD were decreased, and the expression and activities of the oxidase NADPH P67 and gp91 were increased in PE-stimulated cardiomyocytes. The activity of Gpx was also decreased in PE-stimulated cardiomyocytes. Zileuton treatment ameliorated the increased oxidative stress, as evidenced by reduced ROS levels, increased expression and activities of SOD and Gpx, and reduced expression and activity of NADPH oxidase (Figures 4(a)–4(e)).

### 3.5. 5-LOX Silencing Relieves Cardiomyocyte Hypertrophy

**Figure 5:**
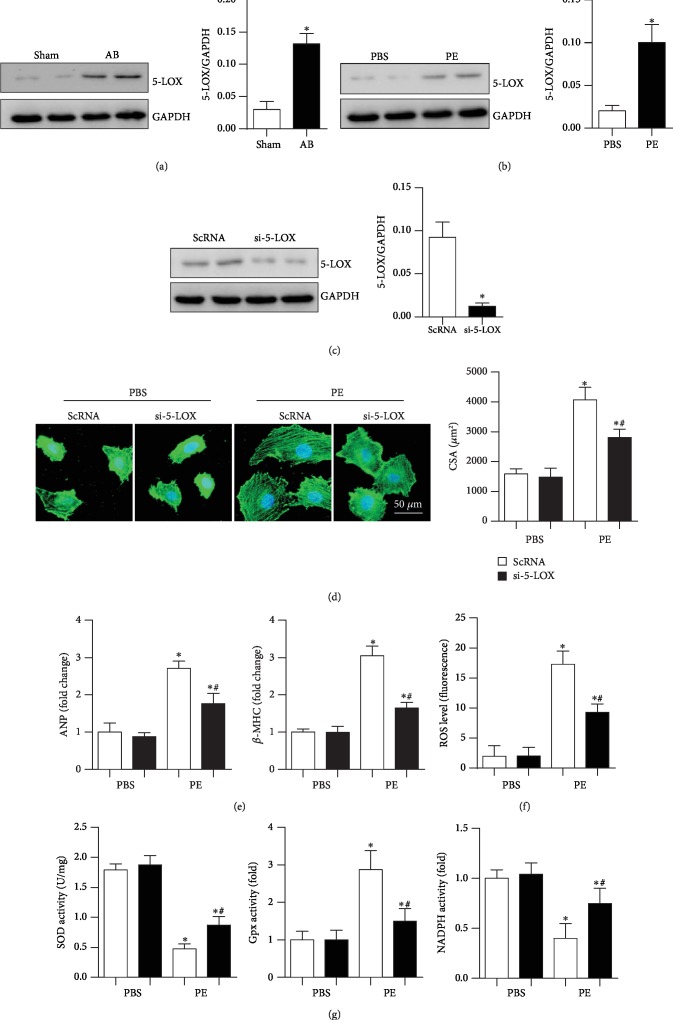
5-LOX silencing relieves cardiomyocyte hypertrophy. (a) Expression level of 5-LOX in heart tissue at 8 weeks after AB surgery (*n* = 6, ^∗^*P* < 0.05 vs. the sham group). (b) Expression level of 5-LOX in cardiomyocytes 24 h after PE stimulation (*n* = 6, ^∗^*P* < 0.05 vs. the control group). (c–g) Cells were transfected with 5-LOX siRNA for 8 h and then stimulated with PE for 24 h. (c) Expression level of 5-LOX after transfection with 5-LOX siRNA (*n* = 6). (d) *α*-Actinin staining (*n* = 6) and quantification of cells. (e) Transcription levels of hypertrophic markers in H9c2 cells (*n* = 6). (f) ROS levels (*n* = 6). (g) Activities of SOD, Gpx, and NADPH oxidase (*n* = 6). ^∗^*P* < 0.05 vs. the ScRNA-PBS group; ^*#*^*P* < 0.05 vs. the ScRNA-PE group.

The expression level of 5-LOX was detected in heart tissue and cardiomyocytes under insult conditions. As shown in Figures 5(a) and 5(b), the expression level of 5-LOX was increased in the pressure overload heart tissue and PE-stimulated cardiomyocytes. Thus, 5-LOX siRNA was used to knockdown 5-LOX (Figure 5(c)) to exclude the effects of zileuton that go beyond the inhibition of 5-LOX. As expected, the cell hypertrophic response induced by PE stimulation was relieved by 5-LOX siRNA, as shown by the reduced cell surface area and reduced ANP and *β*-MHC mRNA levels (Figures 5(d) and 5(e)). Additionally, the increase in oxidative stress induced by PE was reduced by 5-LOX knockdown (Figures 5(f) and 5(g)).

### 3.6. Zileuton Regulates PPAR*α*/NRF2 Signaling

**Figure 6:**
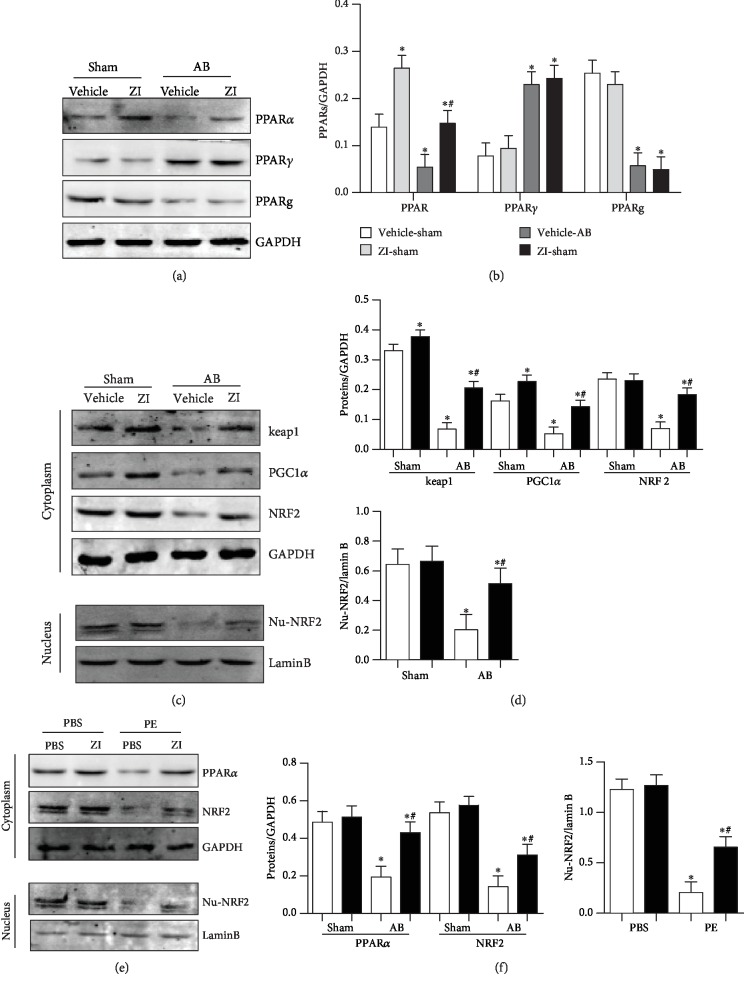
Zileuton regulates PPAR*α*/NRF2 signaling. (a, b) Protein expression levels of PPAR*α*, PPAR*γ*, and PPAR*ƍ* in mouse hearts 8 weeks after AB surgery and 7 weeks after zileuton administration (100 mg/kg/d, orally) (*n* = 6). (c, d) Protein expression levels of keap1, PGC1-*α*, NRF2, and nuclear-NRF2 in mouse hearts (*n* = 6). ^∗^*P* < 0.05 vs. the vehicle-sham group; ^*#*^*P* < 0.05 vs. the vehicle-AB group. (e, f) Protein expression levels of PPAR*α*, NRF2, and nuclear-NRF2 in H9c2 cells (*n* = 6) after 24 h of PE (50 *μ*M) stimulation and zileuton (100 *μ*M) treatment ^∗^*P* < 0.05 vs. the control group; ^*#*^*P* < 0.05 vs. the PE group.

A study reported that zileuton regulated PPAR*γ* signaling in macrophages [12]; thus, we detected oxidative-related PPAR signaling. As shown in Figure 5, PPAR*α* and PPAR*ƍ* expression was decreased and PPAR*γ* expression was increased in AB surgery mouse hearts; zileuton treatment increased only the PPAR*α* expression level and had no significant effects on PPAR*γ* and PPAR*ƍ* expression. Furthermore, the decreased expression levels of the downstream targets keap1 and PGC1*α* and the nuclear translocation of NRF2 in mouse hearts induced by AB surgery were increased by zileuton treatment (Figures 6(a)–6(d)). These phenomena were confirmed in an in vitro study where we found that PE reduced the expression of PPAR*α* and the downstream targets keap1, PGC1*α*, and NRF2; PE also reduced the nuclear translocation of NRF2 in cardiomyocytes, while zileuton treatment blunted these changes (Figures 6(e) and 6(f)).

### 3.7. Blocking PPAR*α*/NRF2 Signaling Counteracts Zileuton's Effect In Vitro

**Figure 7:**
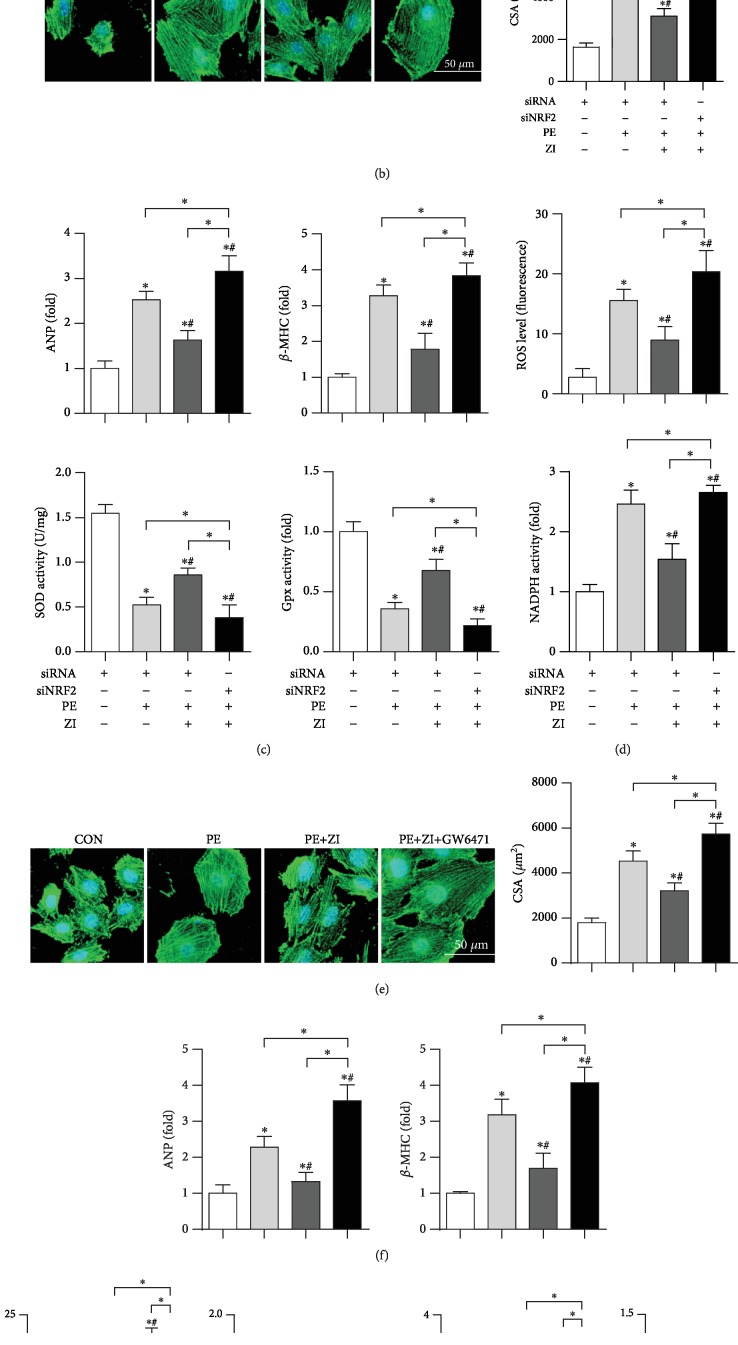
Blocking PPAR*α*/NRF2 signaling counteracts zileuton's effect on vitro. (a–c) Cells were treated with NRF2 siRNA for 8 h and then treated with PE (50 *μ*M) and zileuton (100 *μ*M) for 24 h. (d–g) Cells were treated with PE (50 *μ*M), zileuton (100 *μ*M), and GW6471 (10 *μ*M) for 24 h. (a) Expression of Nrf2 in cells (*n* = 6, ^∗^*P* < 0.05 vs. the control group). (b, e) *α*-Actinin staining (*n* = 6) and quantification results in H9c2 cells. (c, f) Transcription levels of hypertrophic markers (*n* = 6). (d, g) ROS levels and activities of SOD, Gpx, and NADPH oxidase (*n* = 6). ^∗^*P* < 0.05 vs. the control group; ^*#*^*P* < 0.05 vs. the PE group.

NRF2 siRNA was used to knockdown NRF2 in cardiomyocytes (Figure 7(a)). The antihypertrophic effects of zileuton were counteracted by NRF2 knockdown as evidenced by increased PE-induced hypertrophic marker transcription, cell surface area, and oxidative stress (Figures 7(a)–7(d)) when compared with those in the PE group and PE+ zileuton group. Cells were also treated with the PPAR*α* inhibitor GW6471. The antihypertrophic effects of zileuton were also counteracted by GW6471 as indicated by increased PE-induced hypertrophic marker transcription, cell surface area, and oxidative stress (Figures 7(e)–7(g)) when compared with those in the PE group and PE+ zileuton group.

### 3.8. PPAR*α* Knockdown Blunts Zileuton's Antihypertrophic Effect In Vivo

**Figure 8:**
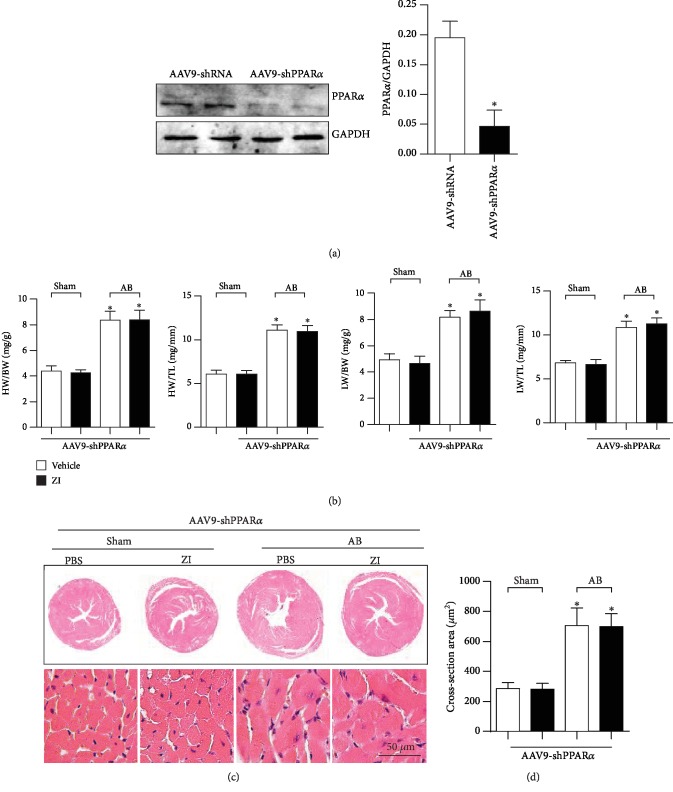
PPAR*α* knockdown blunts zileuton's antihypertrophic effect in vivo. Mice were subjected to AAV9-shPPAR*α* injection. Two weeks after injection, the mice were subjected to AB surgery. One week after AB, the mice were treated with zileuton (100 mg/kg/d) for 7 weeks. (a) PPAR*α* levels in the heart 10 weeks after AAV9-shPPAR*α* injection (*n* = 6). (b) HW/BW, HW/TL, LW/BW and LW/TL ratios in mice (*n* = 12). (c, d) HE staining (*n* = 6) and quantification results in mice (*n* > 50 cells). ^∗^*P* < 0.05 vs. the vehicle-sham group; ^*#*^*P* < 0.05 vs. the vehicle-AB group.

To confirm the central role of PPAR*α* in zileuton's antihypertrophic effect, myocardial injection of AAV9-shPPAR*α* was performed in mice to knockdown PPAR*α* (Figure 8(a)). The knockdown of PPAR*α* completely blunted the antihypertrophic effect of zileuton because the HW/BW, HW/TL, LW/BW, and LW/TL ratios and cell surface area were not significantly different between vehicle-treated mice and zileuton-treated mice subjected to AB (Figures 8(b)–8(d)).

### 3.9. PPAR*α* Knockdown Blunts Zileuton's Antioxidative Effect and Cardioprotection In Vivo

**Figure 9:**
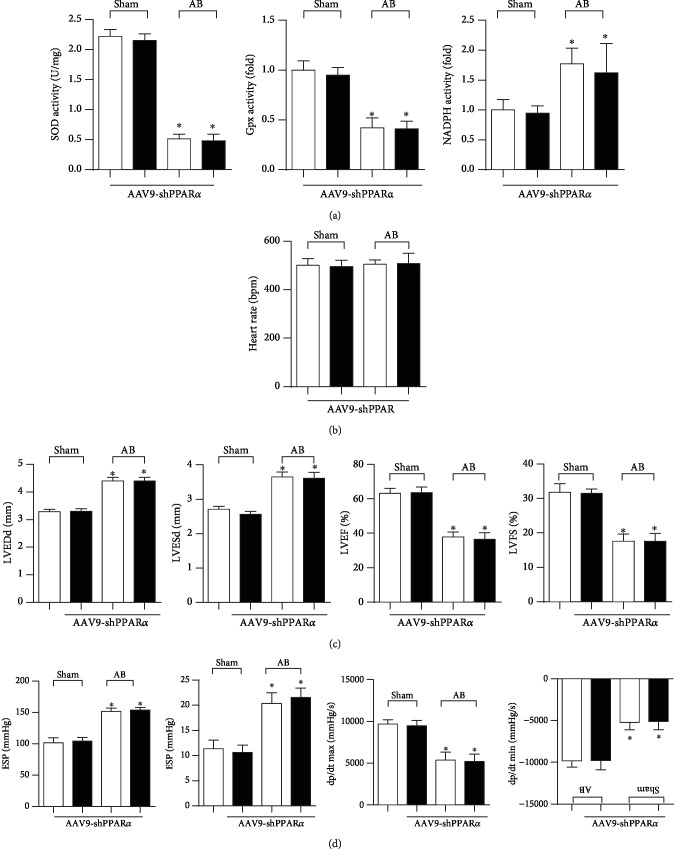
PPAR*α* knockdown blunts zileuton's antioxidative effect and cardioprotection in vivo. Mice were subjected to AAV9-shPPAR*α* injection. Two weeks after injection, the mice were subjected to AB surgery. One week after AB, the mice were treated with zileuton (100 mg/kg/d) for 7 weeks. (a) Activities of SOD, Gpx, and NADPH oxidase in mouse hearts (*n* = 6). (b, c) Echocardiography results (*n* = 10). (d) Hemodynamic measurements (*n* = 10). ^∗^*P* < 0.05 vs. the vehicle-sham group; ^*#*^*P* < 0.05 vs. the vehicle-AB group.

The oxidative effect was evaluated in PPAR*α* knockdown mice in vivo. Excessive oxidative stress was observed in failing mouse hearts as evidenced by decreased antioxidase SOD and Gpx activities and increased oxidase NADPH activity. However, upon PPAR*α* knockdown, these alterations were not significantly different between vehicle-treated and zileuton-treated mouse hearts (Figure 9(a)). LV dilation and dysfunction were also not significantly different between vehicle-treated and zileuton-treated mouse hearts with PPAR*α* knockdown (Figures 9(b)–9(d)).

## 4. Discussion

In this study, zileuton's inhibitory effect against 5-LOX protected mice against stress overload-induced cardiac remodeling. Pharmacological inhibition of 5-LOX prevented cardiac inflammation and excessive oxidative stress, thus improving cardiac function. Mechanistically, 5-LOX inhibition in mice induced the expression of PPAR*α*, but not PPAR*γ* or PPAR*ƍ*. This protective effect of 5-LOX inhibition occurred directly in cardiomyocytes and was related to ROS production reduction. PPAR*α* knockdown in mice counteracted the protective effect of zileuton.

Stimulated by physiology, pharmacology, and pathology, AA is one of the major fatty acids that is released from membrane lipids. Three enzyme systems can metabolize free AA: cyclooxygenase (COX), LOX, and cytochrome P-450 (CP450), which produce prostaglandins (PGS), LTS, epoxyeicosatrienoic acid (EETS), and hydroxyeicosatrienoic acid (HETES) [17]. In the past two decades, studies have focused on PGs and COX in determining the biological significance of AA and its metabolites. The effects of LTs on the cardiovascular system are now established [18, 19]. Both LTs and cysLTs are reported to be increased in failing hearts and contribute to the progression of heart failure [20–22]. Thus, inhibition of this LOX pathway may be beneficial. In this study, we found that the 5-LOX inhibitor zileuton ameliorated pressure overload-induced cardiac hypertrophy and inflammation and improved cardiac function. These data indicate the beneficial role of 5-LOX inhibition and are consistent with a previous report that 5-lipoxygenase inhibition by zileuton protects the heart against ischemia/reperfusion injury [8]. Interestingly, we found that 5-lipoxygenase inhibition by zileuton could directly protect against cardiomyocyte hypertrophy in response to PE in vitro. These results suggest that the protective effect of 5-LOX inhibition may be independent of exogenous LTs, which directly affect cardiomyocytes responding to PE. These findings are also consistent with Kwak HJ's study that zileuton protected against H_2_O_2_-induced cell death in cardiomyocytes [8].

It is believed that the production of ROS is a form of pathological cellular stress but the synthesis and degradation of ROS are now considered to be physiological and steady-state functions of many cells [23]. However, once ROS levels cannot be balanced through appropriate production and removal regulation, oxidative stress may occur and excess ROS will cause protein, DNA, and lipid modifications [1]. Oxidative stress is increased in many pathological processes, including HF, hypertension, cardiac fibrosis, and hypertrophy, and is highly relevant to the development of various cardiovascular diseases [24]. In addition to resulting in cardiomyocyte death, ROS activate a variety of hypertrophic signaling kinases and transcription factors that contribute to cardiac remodeling and heart failure [1]. Interventional studies using a variety of “antioxidant” approaches have been shown to ameliorate LV remodeling [16]. A previous study reported that 5-LOX inhibition could suppress the production of ROS in many cell types [25, 26]. In this study, the 5-LOX inhibitor zileuton attenuated lipid peroxidation, reduced NADPH oxidase expression and activity, and increased SOD expression and activities both in vivo and in vitro. These data indicate that zileuton not only reduced ROS production but also regulated antioxidase and oxidase expression levels.

PPAR*α* is vital in the regulation of fatty acid oxidation, which regulates adipogenesis, lipid and glucose metabolism, and inflammation pathways [27]. PGC-1*α* can bind directly to PPARs that regulate its transcriptional regulatory function via downstream transcriptional regulatory factors, such as NRF2 [4]. Many cytoprotective and detoxifying genes are regulated by NRF2, which can bind to antioxidant response elements (AREs) [28]. Due to decreased antioxidant capacity in the heart, PPAR*α* knockout mice showed increased mitochondria abnormalities leading to increased oxidative stress production, cardiac dysfunction, and cardiac structural changes [29, 30]. A previous study reported that in apoptotic macrophages, 5-LOX can activate PPAR*γ* [12]. However, Pu et al. found that PPAR*α* was activated in 5-LOX-deficient mice [31]. In our study, we found that 5-LOX inhibition in cardiomyocytes causes the activation of PPAR*α*, but not PPAR*γ* or PPAR*ƍ*. This activation by zileuton mediated its cardioprotective effects, which was confirmed by PPAR*α* knockdown in vivo. The activation of PPAR*α* in zileuton-treated mice and cardiomyocytes is intriguing. Various natural fatty acids and eicosanoids are also PPAR ligands [4]. During AA metabolism, PPAR*α* can be activated by LTB4 and 8(S)-HETE and PPAR*γ* can be activated by 12(S)-HETE and 15(S)-HETE [31]. Zileuton inhibition of 5-LO might reduce the endogenous ligands of PPAR*α*, and this hypothesis is supported by a recent study showing that a 5-LOX inhibitor increased PPAR*α* activation [32].

In our study, we used 1, 10, 50, and 100 *μ*M zileuton in vitro and 100 mg/kg/d zileuton (orally) in vivo. We found a dose-dependent antihypertrophic effect of zileuton on the in vitro study, and we chose 100 *μ*M for the other in vitro experiments. Further study of whether the dosage used in vitro (100 *μ*M) corresponds to the in vivo drug dosage (100 mg/kg/d, 7 weeks, oral administration) and whether the dose used in our in vivo experiment could facilitate a stable concentration in the blood and heart tissue should be conducted to find the most efficient drug delivery method and dosage for the treatment of HF.

In summary, 5-LOX inhibition by zileuton protects against cardiac hypertrophy, inflammation, and dysfunction in response to LV pressure overload by activating PPAR*α*/NRF2 signaling in cardiomyocytes, which mediates oxidation balance.

## 5. Clinical Perspectives

1. Zileuton protected mice against cardiac hypertrophy, fibrosis, and oxidative stress in response to LV pressure overload by activating PPAR*α*/NRF2 signaling in cardiomyocytes
2. These results confirm the role of 5-LOX in cardiac remodeling and the usage of a 5-LOX inhibitor (zileuton) for treating heart failure

## Figures and Tables

**Table 1:** Echocardiographic and hemodynamic data measured 1 week after AB before the start of zileuton treatment.

	Vehicle-AB (*n* = 8)	Zileuton-AB (*n* = 8)
LVEDD (mm)	3.3 ± 0.13	3.26 ± 0.10
LVESD (mm)	2.63 ± 0.17	2.64 ± 0.18
LVEF (%)	71.4 ± 6.36	70.6 ± 5.87
LVFS (%)	31.4 ± 2.20	32 ± 3.5
ESD (mmHg)	140.1 ± 4.8	139.3 ± 4.0
EDD (mmHg)	10.3 ± 1.2	9.6 ± 1.8
dp/dt max (mmHg/s)	9525 ± 554	9491 ± 1255
dp/dt min (mmHg/s)	−9031 ± 557	−9191 ± 452

## Data Availability

Data will be available upon request from the corresponding author.
